# Breast Cancer Incidence in Black and White Women Stratified by Estrogen and Progesterone Receptor Statuses

**DOI:** 10.1371/journal.pone.0049359

**Published:** 2012-11-14

**Authors:** Michael X. Gleason, Tengiz Mdzinarishvili, Simon Sherman

**Affiliations:** Eppley Cancer Institute, University of Nebraska Medical Center, Omaha, Nebraska, United States of America; Indiana University, United States of America

## Abstract

**Background:**

There is increasing evidence that breast cancer is a heterogeneous disease presented by different phenotypes and that white women have a higher breast cancer incidence rate, whereas black women have a higher mortality rate. It is also well known that white women have lower incidence rates than black women until approximately age 40, when rate curves cross over and white women have higher rates. The goal of this study was to validate the risk of white and black women to breast cancer phenotypes, stratified by statuses of the estrogen (ER) and progesterone (PR) receptors.

**Methodology/Principal Findings:**

SEER17 data were fractioned by receptor status into [ER+, PR+], [ER−, PR−], [ER+, PR−], and [ER−, PR+] phenotypes. It was shown that in black women compared to white women, cumulative age-specific incidence rates are: (i) smaller for the [ER+, PR+] phenotype; (ii) larger for the [ER−, PR−] and [ER−, PR+] phenotypes; and (iii) almost equal for the [ER+, PR−] phenotype. Clemmesen's Hook, an undulation unique to women's breast cancer age-specific incidence rate curves, is shown here to exist in both races only for the [ER+, PR+] phenotype. It was also shown that for all phenotypes, rate curves have additional undulations and that age-specific incidence rates are nearly proportional in all age intervals.

**Conclusions/Significance:**

For black and white women, risk for the [ER+, PR+], [ER−, PR−] and [ER−, PR+] phenotypes are race dependent, while risk for the [ER+, PR−] phenotype is almost independent of race. The processes of carcinogenesis in aging, leading to the development of each of the considered breast cancer phenotypes, are similar in these racial groups. Undulations exhibited on the curves of age-specific incidence rates of the considered breast cancer phenotypes point to the presence of several subtypes (to be determined) of each of these phenotypes.

## Introduction

Breast cancer (BC) is the most common malignancy diagnosed in women in the United States with about 40,000 women dying of this disease annually [1]. Incidence and mortality are different among racial groups, with a higher incidence rate for white women, whereas African American women are dying from breast cancer at higher rates than whites [1]. Lifestyle factors, such as having no children [2] or having them later in age [3], as well as the use of oral contraceptives [4], hormone replacement therapy [5], or alcohol [6], low or no amount of breastfeeding [7], and being overweight/obese [8] are all associated with the increased risk of breast cancer occurrence.

Age is a common risk factor for majority of cancers, and it is widely accepted that carcinogenesis in aging is coded in the pattern of the age-specific incidence rates [9]. For many cancer types, their rates are graphically exhibited as having low values at young age, sharply increasing values in middle age, and then the values of rates are leveling and declining near the age of 70–80 [10]. When the curves of the age-specific incidence rates do not have inflection points, it is often assumed that the population at risk of getting cancer can be presented as a homogeneous distribution and that the size of the subpopulation that eventually will get cancer is dependent on other, not fully discovered yet, risk factors. Such a simplified assumption has helped researchers to model carcinogenesis in aging for several organ-specific sites [11]–[13].

However, in contrast to other types of cancer, the curve of the breast cancer age-specific incidence rate for women has a unique undulation occurring near the age of 50, called Clemmesen's Hook [14], that has been hypothesized to be associated with menopause [15]–[17]. Presence of Clemmesen's Hook allowed some researchers to assume that breast cancer is a heterogeneous disease with different etiologies (breast cancer phenotypes) and that the population of breast cancer patients can be fractioned by their exposure to distinct risk factors. However, epidemiological characterization of breast cancer subpopulations (fractions) that during the human lifetime are differentially exposed to distinct (but yet to be elucidated) risk factors associated with occurrence of different breast cancer phenotypes remains a challenge.

In oncology, the idea of heterogeneity of breast cancer population is not new. For years, clinicians have divided breast cancer patients into two groups by age at diagnosis, as having two different diseases [17]–[20]. The first group, called early onset, is formed mainly from the patients diagnosed with breast cancer at younger ages, with incidence peaking in this subgroup at an age near 40, while the second group, called late onset, is diagnosed with breast cancer at older ages and is characterized with incidence peaking at an age near 70. The age of menopause, which is approximated by the age of 50, is often used as a simple cut-point in many clinical trials [21]–[24], and serves as a boundary between early and late onset patients.

Separation of breast cancer population into two fractions by a cut-point age, however, may reflect only an overall tendency of the breast cancer phenotypes to be prevalent at extreme ages [17]. A single cut-point solution is an oversimplification of the problem of classification of breast cancer subpopulations, because such an approach does not account for a possibility that the breast cancer age-specific incidence rates, stratified by distinct risk factors and markers, could have unique patterns in aging. On the other hand, current data suggest that breast cancer rates stratified by non-modifiable risk factors, as race, do have different patterns in aging: breast cancer rates in white women are lower than those for black women until approximately age 40, whereupon the curves of breast cancer rates cross over, and from then onward, rates in black women are lower than those for white women [25]–[30].

The existence of this so-called black-to-white ethnic crossover [30], has been documented in [25]–[28], [30], but the reason for the crossover is not completely understood. Some authors argue that the crossover is not a true feature of breast cancer rate curves, instead suggesting that the crossover would disappear after adjusting the rates to account for birth cohort or time period effects [31], [32]. However, age-period-cohort (APC) analysis on age-specific breast cancer incidence rate curves performed in [30] proved that this crossover remains after accounting for birth cohort and time period effects. In other work [28], it was proposed that the race-specific differences can be explained by differences in well-known risk factors (such as older age at first birth, fewer births, younger age of menarche). Although this assumption may explain the disparity between age-specific rates in white and black women after age 40, but it does not explain what is seen in rate curves prior to age 40. Overall, the underlying cause of the crossover remained unknown and the crossover have not been considered yet as additional evidence of breast cancer's biological heterogeneity [29], [33]–[37].

In this work, we assumed that to better understand the nature of the black-to-white crossover, as well as to perform a robust separation of the breast cancer population on fractions with distinct breast cancer phenotypes, there is a need to stratify breast cancer subtypes by biological markers, such as estrogen receptor (ER) status and progesterone receptor (PR) status, which have been shown to be associated with breast cancer development [38]–[40]. This assumption inspired us to analyze breast cancer cases containing information on ER and PR status. For this purpose, we used breast cancer data, collected during the time period of 2004–2008 in the 17 registry set of the National Cancer Institute's Surveillance Epidemiology and End Results (SEER17) database [41], which was the most recent SEER-based breast cancer data (containing information on ER and PR status) available at the time of this work. However, data collected during the five-year time period (2004–2008) are cross-sectional data, containing information on breast cancer patients born in different time periods, and thus could be biased by the birth cohort effects. Because the SEER database includes information on cancer patients for nine registries (SEER9) beginning in 1975, potential birth cohort effects that would preclude analysis of cross-sectional data can be identified. Furthermore, we chose the years 2004–2008 to minimize the potential confounding time period effect of hormone replacement therapy (HRT), whose use was known to have dramatically decreased immediately [42] following the publication of the Women's Health Initiative results regarding HRT in 2002 [43].

Overall goal of this study was to validate the risk of white and black women to breast cancer phenotypes, stratified by the ER and PR statuses.

## Materials and Methods

### Data Preparation

In this study we utilized the SEER registries that accumulate population-based data on patients (cases) diagnosed with different types of cancer during distinct time periods and U.S. Census Bureau data (supplied by SEER) that provide information on the population at risk of getting cancer. From SEER we used data on two breast cancer populations: (*i*) the population of white and black women diagnosed with breast cancer between 1979 and 2008 and documented in the SEER9 registry subset; and (*ii*) the population of white and black women diagnosed with breast cancer between 2004 and 2008 and documented in SEER17. The SEER9 registries contain information on cancer cases collected from the following nine geographical areas: Atlanta, Connecticut, Detroit, Hawaii, Iowa, New Mexico, San Francisco-Oakland, Seattle-Puget Sound, Utah. The SEER17 registries include the SEER9 registries and information on cancer cases collected in eight additional areas: Los Angeles, San Jose-Monterey, Rural Georgia, Greater California, Kentucky, Louisiana, New Jersey, and Alaska. (In our study we did not use data from the Alaska registry, because that registry only contains cases for American Indian/Alaska Natives.) We studied cases only for women known to be black or white, which account for 91.5% of all female breast cancer patients in the SEER database. To determine race, SEER requires participating centers to use all resources, including medical records, face sheets or photographs, and physician or nursing notes. In order to minimize the number of patients classified as unknown race, if race remains unclear, place of birth, nationality, and surname are permitted to ascertain race, but only if these data are accordant and reveal race unambiguously. The remaining 8.5% of cases were for women whose race was coded by SEER as “unknown” (which may include multiracial women) or whose race that was not white or black (which includes Native Americans and Asian Americans/Pacific Islanders). These cases were not studied because they were not available in sufficient quantities to achieve statistical significance.

To analyze potential age-period-cohort (APC) effects on breast cancer occurrence in populations of white and black women, we used data obtained from the SEER9 registries. This is because the longitudinal nature of the APC analysis required utilization of data from much earlier time periods, when only nine registries were available three decades ago. Of 903,804 SEER9 cases of breast cancer in white and black women of known age, we excluded 148,106 cases that were not the first primary cancer. In accordance with the SEER Survival Monograph [44], we excluded cases that were indicative of poor annotation, and thus we excluded 4,150 cases diagnosed by death certificate only or at autopsy, and 4,803 cases lacking follow-up information. We also excluded 114,401 cases of in situ breast cancer and 6,630 cases that were not microscopically confirmed. To prevent possible confounding by childhood cancers, we further excluded 59 cases for patients aged less than 20 years. The remaining 626,045 cases were used to perform APC analysis.

The obtained breast cancer cases and the corresponding population at risk (determined from U.S. Census Bureau data that is provided with the SEER database) were grouped in 5-year cross-sectional time-period groups between 1979–2008; 5-year age groups between 20–84 years; and 5-year groups corresponding to the birth cohorts between 1894-1988. For each age interval, incidence rates were age-adjusted by the direct method [45] using the 2000 United States standard population [46] and expressed per 100,000 women. Below, age-specific incidence rates are denoted as *I*(*t_i_*) (*i* = 1, …, 13).

The breast cancer populations of black and white women presented in SEER17 registries for the time-period of 2004–2008 were fractioned by (positive and/or negative) ER and PR status. We utilized the SEER database fields named “Collaborative Staging Site Specific Factor (CSSSF) 1” and “CSSSF 2,” for ER status and PR status, correspondingly, which are specifically defined for breast cancer starting with the year 2004. Of 289,739 SEER17 cases of breast cancer in white and black women of known age, we excluded 15,113 cases for individuals aged greater than 84 years, 26 cases for individuals aged less than 20 years, 49,499 cases that were not first primary breast cancer, 499 cases diagnosed by death certificate only or at autopsy, 4,336 cases lacking follow-up information, 42,610 cases of in situ breast cancer, 950 cases that were not microscopically confirmed, and 15,500 cases that had indefinite ER or PR status, leaving 161,206 cases for analysis. Receptor status was assumed to be indefinite, when in the case records, it was marked as: “Borderline; undetermined whether positive or negative,” or “Ordered, but results not in chart,” or “Unknown or no information,” or “Not Performed.” The obtained four fractions were denoted as: [ER+, PR+], [ER+, PR−], [ER−, PR+], and [ER−, PR−].

### Data Analysis

The APC analysis was performed by the approach we described previously [47].

The cumulative incidence rates (CIR) of the fractions, which indicate risk for breast cancer phenotypes, were calculated as the sum of the age-specific incidence rates *I*(*t_i_*), over all age intervals, *t_i_*. Standard errors (*SE*) of the cumulative incidence rates were calculated as a square root of sum of squares of standard errors of the *I*(*t_i_*). For black and white women we estimated cumulative incidence rates (and their *SE*s) for the [ER+, PR+], [ER+, PR−], [ER−, PR+], and [ER−, PR−] phenotypes.

In addition, we estimated relative risk of white (*W*) vs. black (*B*) women (denoted as 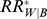) to get a specified breast cancer phenotype using a set of ratios: 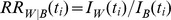, where *I_W_*(*t_i_*) and *I_B_*(*t_i_*) are the age-specific incidence rates at age interval *t_i_*, for black and white women correspondingly. The standard error of the relative risk, *SE*[*RR_W|B_*(*t_i_*)], was estimated by using the *SE*[*I_W_*(*t_i_*)] and *SE*[*I_B_*(*t_i_*)] by the standard rules of error propagation. The estimate of the averaged relative risk, 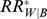, was calculated by the following formula of weighted mean:
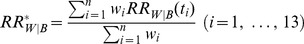
(1)


In Equation (1), the weights *w_i_*, are given as reciprocals of the square of the *SE*[*RR_W|B_*(*t_i_*)]. We calculated the *SE* of the corresponding estimate using the following variance of the weighted mean:
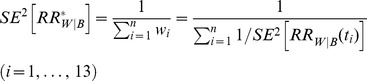
(2)


For each considered breast cancer phenotype, we calculated the corresponding CIR, *SE*s and 95% confidence intervals of the fractions of standardized populations of black and white women, as well as the relative rates and corresponding *SE*s and 95% confidence intervals of black women *vs.* white women, 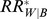.

## Results and Discussion

### Age-Period-Cohort (APC) Effects on Breast Cancer Presentation by Age

**Figure 1:**
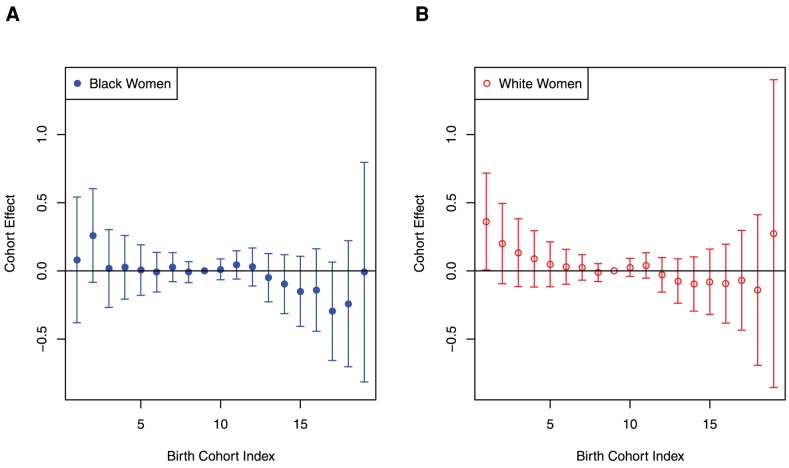
Birth cohort effects (shown in logarithmic scale) on breast cancer rates in black and white women diagnosed in 1979–2008. The effects are calculated by age-period-cohort analysis for cases from the Surveillance Epidemiology and End Results 9 registry database. Cohorts are indexed by 5-year intervals (*i* = 1, 2, …, 19) beginning with 1894–1899 and ending with 1975–1979. Error bars indicate 95% confidence intervals.

To analyze APC effects on breast cancer presentation in aging, we utilized SEER9 data from breast cancer cases in white and black women diagnosed with breast cancer during 1979–2008. Using our previously developed approach, we estimated time period and birth cohort effects and calculated age-specific incidence rates adjusted for these effects [47]. The performed APC analysis showed that in both black and white women, no significant birth cohort effect trends are present (Figure 1), as each cohort's individual effect is not significantly different from zero.

**Figure 2:**
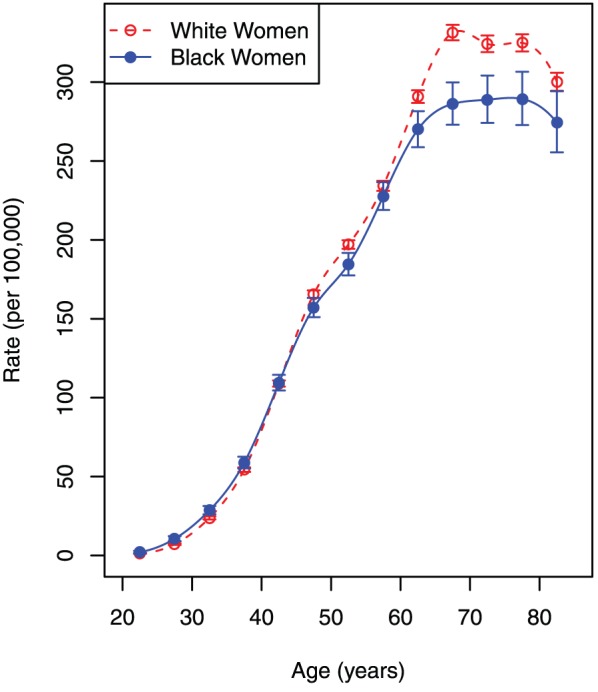
Age-specific incidence rates of breast cancer cases in black and white women diagnosed in 2004–2008. Case data are from the Surveillance Epidemiology and End Results 17 registry database. The black-to-white ethnic crossover is observed near the age of 40, and Clemmesen's Hook can be seen at the 45–49 age interval. Error bars indicate 95% confidence intervals.

Absence of significant birth cohort effects allowed us to reduce our further analysis by utilizing just cross-sectional data for the population of white and black women diagnosed with breast cancer between 2004 and 2008 and documented in SEER17. Age-specific breast cancer incidence rates in white and black women, 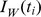 and 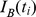, determined for these cross-sectional data are presented on Figure 2. Overall, these rates increase within the age interval of 20–64, flatten within the age interval 65–74, and then decrease at older ages. The undulation known as Clemmesen's Hook [14] is clearly seen in the 45–49 age interval. Rate curves for black and white women also differ in both magnitude and shape, and show that without stratification of breast cancer by ER or PR status, there is no proportionality between values of 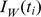 and 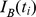 for all age intervals, *t_i_*. These rate curves also exhibit a crossover point near the age of 40: for women younger than 40, 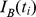 exceeds 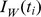, while for older ages, 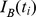 have lower values than 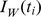.

### Assessment of Risk of White and Black Women to Breast Cancer Phenotypes, Stratified by ER and PR Statuses

**Figure 3:**
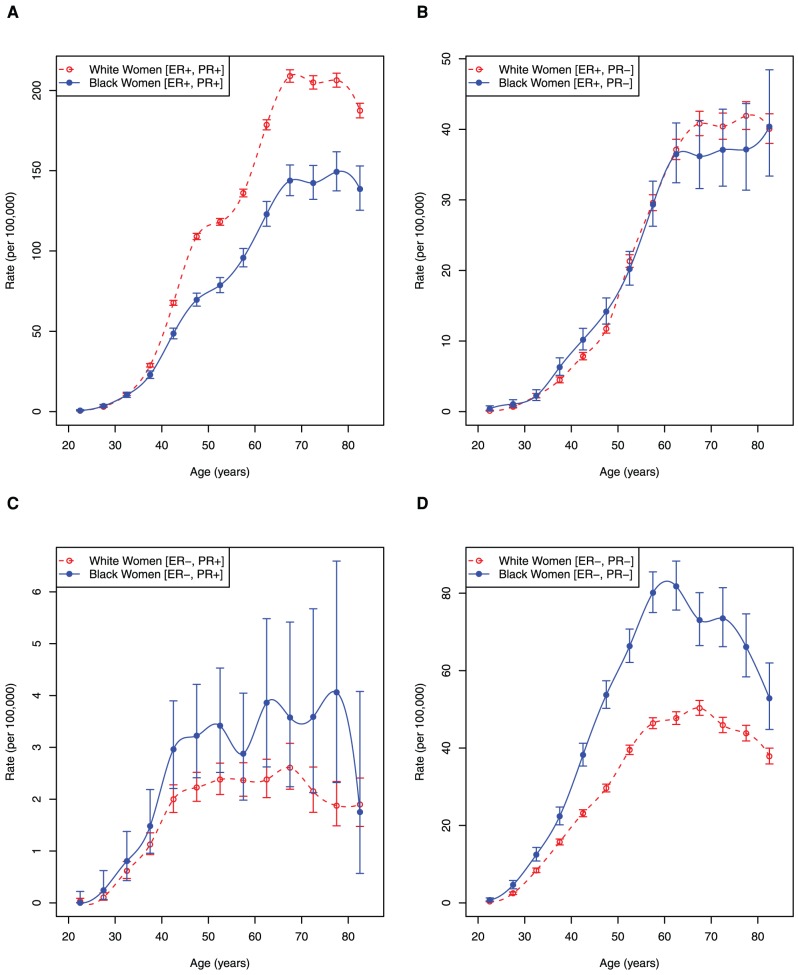
Age-specific incidence rates by breast cancer phenotype in black and white women. Error bars indicate 95% confidence intervals.

The robust detection of the Clemmesen's Hook and uncovering differences in the shapes of 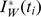 and 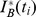, evidenced by the black-to-white ethnic crossover, signifies that breast cancer is a heterogeneous disease comprised of several phenotypes. To elucidate some (presumably important) features of breast cancer age-specific incidence rates, we considered groups (fractions) of breast cancer cases stratified by ER and PR status, resulting in four phenotypes ([ER+, PR+], [ER+, PR−], [ER−, PR+], and [ER−, PR−]) for analysis. For each phenotype we determined the age-specific incidence rate (and *SE*) in white and black women. These rates were denoted *I* ^+,+^(*t_i_*), *I* ^+,−^(*t_i_*), *I* ^−,+^(*t_i_*), and *I* ^−,−^(*t_i_*), correspondingly. The obtained rates are exhibited as Figure 3. Visual inspection of these rates shows that stratification by ER and PR statuses further unmasks the complexity of breast cancer disease and its diversification in black and white ethnic groups.

Comparative analysis of amplitude and morphology of the breast cancer age-specific incidence rates, shown on Figure 3, as well as localization of inflection points and wave-like patterns (undulations) of their curves occurring in the older ages on the age scale highlight this complexity. As it is easy to see (Figure 3A), the values of 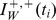 are larger than the values of 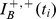 in all age intervals *t_i_*, whereas the values of 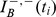 are larger than the values of 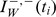 (Figure 3D). For the [ER+, PR−] phenotype the confidence intervals of the 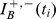 and 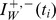 are overlapping (Figure 3B). Figure 3C shows that the values of 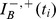 are larger than the values of 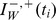 but their confidence intervals are overlapping.

As can be seen from Table 1, the cumulative incidence rate of the [ER+, PR+] phenotype in white women, 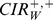, is significantly larger (*z*-test, 2-sided, *p*<0.05; confidence intervals do not overlap) than 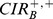 and 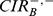 is significantly larger than 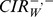. In contrast, there is no significant difference between 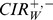 and 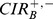. The values for 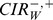 and 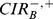 are very small and account for only 1.2% of cases, but 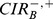 is significantly larger than 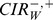. Values of the averaged relative rate occurring in a given breast cancer phenotype in black *vs*. white women, 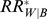, and coefficients of proportionality between the age-specific breast cancer incidence rates of the fractions of standardized populations of black and white women are presented in Table 1.

**Table 1:** Cumulative breast cancer incidence rates by race (*CIR_W_*, *CIR_B_*) and corresponding scaling coefficient (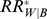).

	Breast Cancer Phenotype							
	[ER+, PR+]		[ER−, PR−]		[ER+, PR−]		[ER−, PR+]	
	Estimate	95%CI	Estimate	95%CI	Estimate	95%CI	Estimate	95%CI
***CIR_W_***	1460.1	(1450.1, 1470.1)	391.3	(386.4, 396.3)	278.3	(273.9, 282.8)	21.8	(20.6, 22.9)
***CIR_B_***	1026.9	(1000.9, 1052.9)	625.9	(607.19, 644.70)	271.2	(257.7, 284.7)	31.8	(27.7, 36.0)
***RR*** ^*^ ***_W_***_**|*B***_	1.42	(1.42, 1.42)	0.60	(0.60, 0.60)	0.96	(0.96, 0.96)	0.67	(0.67, 0.67)

**Figure 4:**
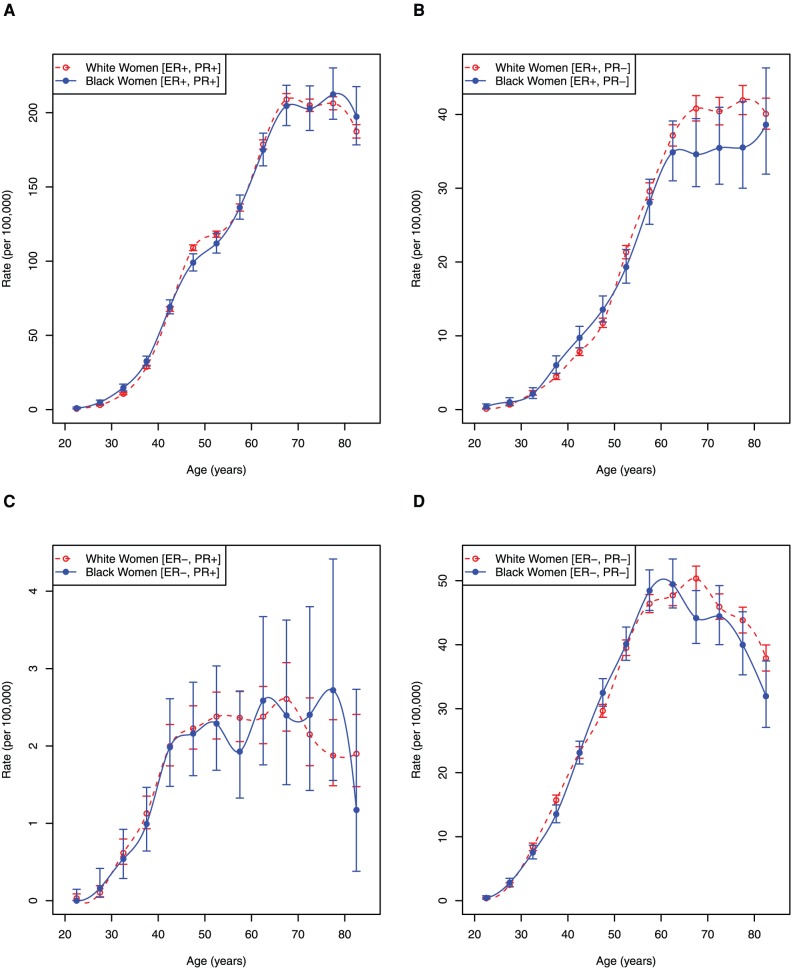
Scaled age-specific incidence rates by breast cancer phenotype in black and white women. Rates for black women (and their error bars) have been scaled by the phenotype-specific coefficient, 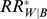 (values shown in Table 1). Error bars indicate 95% confidence intervals.

To better perform visual inspection of proportionality between the age-specific incidence rates of breast cancer phenotypes, stratified by ER and PR status, in black and white women, we adjusted the age-specific incidence rates in black women to the analogous rates in white women by scaling with the corresponding coefficients, 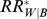, presented in Table 1. Figure 4 exhibits the observed age-specific incidence rates of breast cancer phenotypes in white women and the correspondingly adjusted (scaled) to them the age-specific incidence rates of breast cancer phenotypes in black women. As can be seen in Figures 4A, 4B, 4C and 4D, after adjustments the age-specific incidence rates stratified by ER and PR statuses for black women are indistinguishable from the rates for white women at nearly all age intervals. These observations suggest that race is a nearly proportional hazard of presentation of the breast cancer phenotypes, stratified by ER and PR status. If so, then carcinogenesis in aging, resulting in occurrence of each of the considered breast cancer phenotypes should be similar in white and black women. Nevertheless, as it will be discussed below, despite of overall similarities, there are some race-dependent variations in carcinogenesis in black and white women.

Visual examination of *I* ^+,+^(*t_i_*), *I* ^+,−^(*t_i_*), *I* ^−,+^(*t_i_*), and *I* ^−,−^(*t_i_*) profiles in white and black women (Figure 3) highlights some common, as well as distinguished features characterizing carcinogenesis that result in the development of corresponding breast cancer phenotypes. First, acceleration of these rates begins from the age about 20 and continues to approximately age 60, followed by two undulations in the age interval of 60–75, flattening and then decline. The relative amplitudes of the two waves observed between ages 60–75 vary in a phenotype- and race-dependent manner. Interestingly, the phenotypes' age-specific rates have some convexity near the age of menopause, which are clearly visualized as an undulation (or Clemmesen's Hook) on *I* ^+,+^(*t_i_*) for the age 45–49 interval (Figure 3A) but almost completely abrogated on *I* ^+,−^(*t_i_*), *I* ^−,+^(*t_i_*), and *I* ^−,−^(*t_i_*) (Figure 3B–D).

The reasons causing Clemmesen's Hook (for the [ER+, PR+] phenotype) and the other observed undulations (for all considered breast cancer phenotypes) on the curves of the age-specific incidence rates of the fractions of standardized populations of black and white women at risk for the corresponding breast cancer phenotypes, remain unclear. However, these phenomena point to the presence of several subtypes of each of the considered breast cancer phenotypes, and contribute to the growing body of evidence to the heterogeneity of breast cancer. The widely-publicized reports from Perou and colleagues [48], [49] established that breast cancer can be classified by microarray expression profiles (Luminal A, Luminal B, HER2-overexpressing, Basal-like, and Normal-like) and that these subtypes are likely indicative of separate disease types within the umbrella term, “breast cancer.” Recently, another group proposed that breast cancer can be classified into at least 10 subtypes [50], further balkanizing the disease. Our results presented in this work provide evidence for breast cancer heterogeneity via a different route, using analysis of age-specific incidence rates, rather than microarray gene expression profiling. Nevertheless, we reached a similar conclusion, that breast cancer is not a single disease, nor is it clearly demarcated by ER or PR status, and that within a specific [ER, PR] phenotype, multiple subtypes may be present.

Because our data has shown that there is no statistically significant black-to-white crossover after stratifying by ER and PR status, we now know that black women have higher rates of [ER−, PR−] disease regardless of age, and lower rates of [ER+, PR+] disease regardless of age. Researchers may now focus their energies on explaining these race-specific disparities without concern that the disparities reverse themselves in younger ages.

There are several potential limitations of this study. Our study has similar limitations as many other SEER-based studies, which assume that SEER cases represent the United States as a whole and that there is little regional variation in diagnostic and reporting standards for breast cancer case data. In addition, SEER data lacks information on other breast cancer biomarkers, most notably, HER2. These biomarkers could account for unexplained features of breast cancer incidence rate curves, and may exhibit differential presentation when stratified by race. Another concern is that we included [ER−, PR+] in our study, despite there being considerable doubt in the field of the true existence of this phenotype [51], with the hypothesis that these cases are false negatives for ER or false positives for PR. Because there were a small, but significant number of cases (1990, accounting for 1.2% of all cases) classified as [ER−, PR+], we think that it would be inappropriate to exclude these data. We also assume that ER and PR assay methodology and interpretation have not significantly changed within 2004–2008.

Nevertheless, the use of SEER data allowed us to study breast cancer incidences from a population-based perspective, with numbers of cases that are several orders of magnitude higher than many other breast cancer studies. Large numbers of cases provide not only statistical power, but enable use of novel analytical methods, such the age-period-cohort analysis that we utilized to demonstrate lack of birth cohort effects in breast cancer incidences during 1979–2008. Another elegant technique allowed us to quantitatively compare sizes of fractions of age-standardized subpopulations of black and white women diagnosed with breast cancer by utilizing cumulative incidence rates and their standard errors. Also, using qualitative methods, we demonstrated Clemmesen's Hook is only present in the [ER+, PR+] phenotype and the black-to-white crossover depend on ER and PR status. Thus, our findings do not support an assertion made in the recently published work [52] that tumor grade plays the primary role in the heterogeneity of breast cancer age patterns, while the hormone receptor status is only associated with the “remaining” heterogeneity. In fact, in the present work we have demonstrated that accounting for ER and PR statuses alone is sufficient to reveal some important heterogeneous features of breast cancer age patterns.

## Conclusion

In this work, we analyzed how race, as a non-modifiable categorical factor, influences on heterogeneity of sizes of fractions in black and white women at risk for breast cancer phenotypes, stratified by the positive and/or negative statuses of the ER and PR receptors and how race can influence carcinogenesis leading to the development of these breast cancer phenotypes.

We used breast cancer data from the SEER17 registries during the years 2004–2008 and showed that these cross-sectional data are not confounded by birth cohort effects. This was done using APC analysis of longitudinal data collected during the years 1978–2008 in the SEER9 registries. We showed that within this 30 year period birth cohort effects have very little influence on breast cancer age-specific incidence rates. Thus we conclude that birth cohort effects cannot perturb results of this study in which cross-sectional data were utilized.

Using SEER17 breast cancer cases in black and white women for the years 2004–2008, we showed that in these racial groups, sizes of fractions at risk for the considered breast cancer phenotypes are disproportional. In both racial groups, the [ER+, PR+] was the most prevalent breast cancer phenotype. However, for cumulative incidence rates in the fraction of population within the standardized population (comprised from 100,000 individuals in each of five-year long age intervals of human lifespan) at risk for this phenotype in white women, 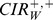, was larger than 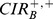. The reverse phenomenon took place for the fractions of black and white women at risk for the [ER−, PR−] phenotype. In both racial groups, their fractions of population at risk for this phenotype had the second largest sizes, but 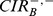 was larger than 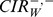. Fractions of populations of black and white women at risk for the [ER+, PR−] phenotype had the third largest sizes, which were statistically indistinguishable in these racial groups. Finally, fractions of populations of black and white women at risk for the [ER−, PR+] phenotype are very small, but the size of this fraction is larger in black women than white women.

Analysis of age-specific incidence rates of the considered breast cancer phenotypes in black and white women showed that in all age intervals, the morphology of the curves of the rates of the corresponding breast cancer phenotypes are similar and that, in most of the age intervals, the rates of a given breast cancer phenotype in black women can be well-adjusted to the corresponding rates in white women by multiplication by corresponding constants, which were also determined in this work.

We have shown that black women have lower risk for the [ER+, PR+] phenotype compared to white women, but higher risk for the [ER−, PR−] and [ER−, PR+] phenotypes. In these racial groups, the risk for the breast cancer with the [ER+, PR−] phenotype was almost the same. It is likely that risks for the breast cancer phenotypes, stratified by ER and PR status, are derived from both hereditary and environmental factors. The obtained results also suggest that carcinogenesis in aging, leading to the development of each of the considered breast cancer phenotypes, is similar in white and black women.

Clemmesen's Hook (for the [ER+, PR+] phenotype) and other undulations (for all breast cancer phenotypes, stratified by ER and PR statuses) observed on the corresponding age-specific incidence rates indicate that each of the considered breast cancer phenotypes is also a heterogeneous disease and suggests the existence of several subtypes of each of these phenotypes. For instance, such subtypes could differ by the status of HER2, which is known to play an important role in breast cancer carcinogenesis [40] or statuses of other potential breast cancer biomarkers (such as Ki67, Cyclin D1, Estrogen Receptor β [53]). Because SEER registries do not provide such data at present, additional data collection efforts are required to further promote epidemiological studies of breast cancer.
